# Mechanochemical Coupling in the Myosin Motor Domain. II. Analysis of Critical Residues

**DOI:** 10.1371/journal.pcbi.0030023

**Published:** 2007-02-16

**Authors:** Haibo Yu, Liang Ma, Yang Yang, Qiang Cui

**Affiliations:** 1 Department of Chemistry, University of Wisconsin, Madison, Wisconsin, United States of America; 2 Theoretical Chemistry Institute, University of Wisconsin, Madison, Wisconsin, United States of America; University of Arizona, United States of America

## Abstract

An important challenge in the analysis of mechanochemical coupling in molecular motors is to identify residues that dictate the tight coupling between the chemical site and distant structural rearrangements. In this work, a systematic attempt is made to tackle this issue for the conventional myosin. By judiciously combining a range of computational techniques with different approximations and strength, which include targeted molecular dynamics, normal mode analysis, and statistical coupling analysis, we are able to identify a set of important residues and propose their relevant function during the recovery stroke of myosin. These analyses also allowed us to make connections with previous experimental and computational studies in a critical manner. The behavior of the widely used reporter residue, Trp501, in the simulations confirms the concern that its fluorescence does not simply reflect the relay loop conformation or active-site open/close but depends subtly on its microenvironment. The findings in the targeted molecular dynamics and a previous minimum energy path analysis of the recovery stroke have been compared and analyzed, which emphasized the difference and complementarity of the two approaches. In conjunction with our previous studies, the current set of investigations suggest that the modulation of structural flexibility at both the local (e.g., active-site) and domain scales with strategically placed “hotspot” residues and phosphate chemistry is likely the general feature for mechanochemical coupling in many molecular motors. The fundamental strategies of examining both collective and local changes and combining physically motivated methods and informatics-driven techniques are expected to be valuable to the study of other molecular motors and allosteric systems in general.

## Author Summary

Molecular motors are inherently allosteric in nature because the small structural changes associated with the chemistry in the active site are propagated over a long distance and amplified into much larger conformational transitions. A fundamental challenge for understanding such processes concerns the identification of residues and interactions that dictate the propagation of the “mechanochemical coupling signals.” By combining a range of computational techniques based on different principles and assumptions, we have made a systematic attempt to identify these hotspot residues in the molecular motor myosin. Although each method has its limitations, the results from different analyses complement and reinforce each other, which makes it possible to meaningfully compare the results here to previous computational and experimental studies. Combined with results from the preceding paper [24], the current set of investigations suggests that the modulation of structural flexibility at both the local (e.g., active-site) and domain scales with strategically placed “hotspot” residues and phosphate chemistry is likely a general feature for many molecular motors. The study also highlights the value of examining both collective and local changes as well as combining physically motivated methods and informatics-driven techniques for analyzing allosteric systems in general.

## Introduction

Large-scale conformational transitions driven by a distant chemical event, such as ligand binding, is the hallmark of many allosteric systems [1]. Although allostery is historically defined for multisubunit systems [2,3], many of which have a considerable degree of symmetry [4], it is increasingly recognized that coordination between events occurring at distant sites is implicated in the function of many systems that are either single-subunit or without any obvious symmetry [5,6]. In this regard, molecular motors are striking examples [7–9], in which the hydrolysis of ATP induces large-scale structural rearrangements in regions far from the nucleotide binding site. Since the function of molecular motors is likely dependent on a tight coupling between the chemical and the mechanical (conformational) events, understanding the mechanism of such “mechanochemical” coupling at the molecular level has been an actively pursued objective in modern biophysics [7,10,11]. Due to the involvement of processes of different length scales and physicochemical nature, however, a detailed mechanistic description remains elusive for most molecular motors and, indeed, allosteric systems in general.

A specific challenge that has both fundamental and practical importance in this context is the identification of key residues that dictate the coupling between distant sites. In molecular motors, these “hotspot” residues ensure the tight coupling between ATP hydrolysis and the mechanical events and therefore are of major functional importance; mutation of such residues will likely lead to “decoupling mutants” [12,13], in which hydrolysis activity is not affected but the mechanical function is compromised or even abolished. Employing a computational approach for identifying these “hotspot” residues is desirable considering the potential efficiency. However, only limited efforts have been made in this regard for allosteric systems in general [6,14], and even less systematic work has been done on molecular motor systems due to their typically large size [15,16].

In the current work, we make an attempt to tackle this issue for a specific molecular motor system, the conventional myosin (thereafter referred to as myosin) [17]. This is motivated by several considerations. Myosin is a prototypical molecular motor; therefore, insights and computational protocols established for myosin might be transferrable to other molecular motor systems. In addition, there is a rich experimental background for myosin [18–23], including diverse structural, mutagenesis, and spectroscopic and motility characterizations, which makes it possible to extensively validate the computational analyses.

**Figure 1:**
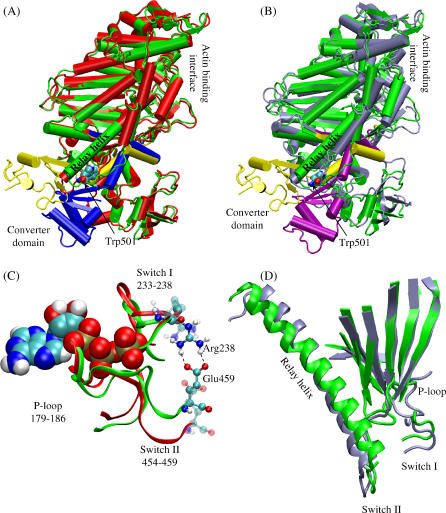
Structural Differences between Conformations of the Dictyostelium discoideum Myosin Motor Domain (A) The difference between the post-rigor (1FMW [29]) and pre-powerstroke (1VOM [30]) states. The structures are aligned based on backbone atoms in the first 650 residues; red and blue correspond to 1FMW, green and yellow correspond to 1VOM. Trp501 is shown in the van der Waals form. (B) The difference between the pre-powerstroke (1VOM [30]), and near-rigor (1Q5G [37]) states. The structures are aligned based on backbone atoms in the first 650 residues; green and yellow correspond to the 1VOM state, slate blue and purple correspond to the 1Q5G state. (C) Superposition of the active site region in 1FMW and 1VOM (same color coding as in (A); the nucleotide is shown in the van der Waals form). (D) Superposition of the central β sheet in 1VOM and 1Q5G (same color coding as in (B); the two structures are superimposed according to the 6th and 7th β strands). The Switch II in the two states are in very similar positions. The distal end of the β sheet (strand 1–4) twists. Note that this leads to a displacement of the P-loop.

As discussed in detail in the accompanying paper [24], the “mechanochemical coupling” in myosin concerns the tight coupling between ATP hydrolysis and structural rearrangements in the converter domain and the actin binding interface, both distant from the nucleotide binding site (see Figure 1). The simulation analysis in the accompanying paper [24] and our previous studies [25,26] established that the coordination between the chemical (hydrolysis) and mechanical (conformational) events is the consequence of structural couplings between the active site and the distant functional sites (i.e., converter and actin-binding interface). Although these structural couplings have been explicitly illustrated [24,26] and even energetically quantified for the coupling between the open/close transition of the active site and changes in the converter domain/relay helix, the residues that dictate these couplings remain to be systematically explored, and this is done here.

Considering the level of complexity, we argue that the best avenue is to combine different computational methods since they provide complementary information regarding the problem at hand. Specifically, targeted molecular dynamics (TMD) simulations [27,28] are used to explore transition pathways between the two conformational states of the motor domain (PDB codes 1FMW [29] and 1VOM [30]), which complements the recent minimum energy path (MEP) analysis of Fischer and co-workers [31]. As alternative ways of systematically identifying important residues to the mechanochemical coupling, normal mode analysis [32,33] and statistical coupling analysis (SCA) [14,34] also are carried out. Since these computational approaches are based on very different principles and assumptions, combining them synergetically has allowed us to make connections with previous experimental and computational analyses in a critical manner; indeed, the interpretation of many experimental probes on such complex systems is not without complications [35]. A series of residues as important regulatory elements for the mechanochemical coupling have been proposed for experimental verification.

## Results

As in the accompanying paper [24], we summarize the nomenclature and conformational characteristics for key structural motifs in Table 1 following the discussions in previous structural studies [23,36]. Specifically, we refer to the active site with the critical salt bridge (Arg238–Glu459) formed as the *closed* configuration; otherwise, the *open* configuration. In the 1FMW structure, the converter domain is in a *down* position with the relay helix straight, while in the 1VOM structure, the converter domain is in an *up* position with a kink in the relay helix (see Table 1 and Figure1A). To address the correlation between structural flexibility and specific transition, the normal mode and hinge analyses have been done for not only the post-rigor (1FMW), pre-powerstroke (1VOM) states but also for the recently reported near-rigor state (1Q5G) [37], which shows a twisting of the central ß sheet with an open active site (Figure 1D) and a lever arm in the *down* position (Figure 1B).

**Table 1:**
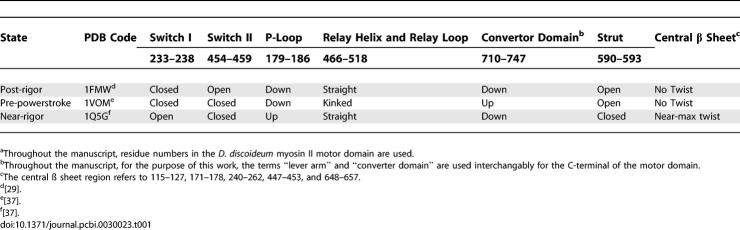
Structural Characteristics for the Three Conformations of the Myosin Motor Domain Characterized with X-Ray Crystallography^a^

### Approximate Transition Path between “Post-Rigor”and “Pre-Powerstroke” Conformational States

To explore the sequence of events along the 1FMW → 1VOM transition (the recovery stroke), three independent TMD simulations have been carried out, and they produce qualitatively rather similar behaviors. The results also allow an analysis of the behavior of Trp501, which has important implications for interpreting the relevant fluorescence-quenching experiments [35,38–40].

#### Sequence of structural changes in TMD simulations.

As mentioned in the Introduction, Fischer and co-workers carried out MEP analysis and proposed a mechanism for the recovery stroke in myosin II (~1FMW → 1VOM) that propagates from the active site into the converter in a highly ordered fashion [31]. The mechanism is consistent with a number of experimental observations (however, see Discussion) and forms the basis for the analysis of the current TMD simulations.

**Figure 2:**
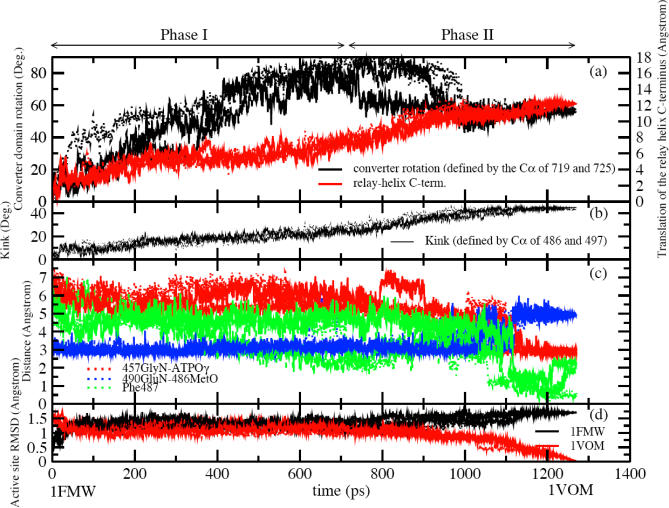
Variation of Critical Structural Parameters Along the Three TMD Trajectories for Illustrating the Sequence of Events Along the Approximation Transition Path between 1FMW and 1VOM (A) Synchronous progress of converter-domain rotation (angle on left axis, black line) and translation of the relay helix C-terminus (distance on right axis, red line). (B) The kink along the relay helix defined by the Cα of Met486 and the Cα of Glu497. (C) Successive local structural reorganizations: (i) formation of the hydrogen bond between Gly457 and the γ-phosphate of ATP (N457-Oγ distance, red line), (ii) breaking of the intrahelical hydrogen bond between Met486 and Glu490 in the relay helix (blue line), (iii) translation of Phe487 (green line). (D) The active site (Switch I, Switch II, and P-loop) backbone atom RMSD values relative to the two reference structures (1FMW and 1VOM) during the transition.

**Figure 3:**
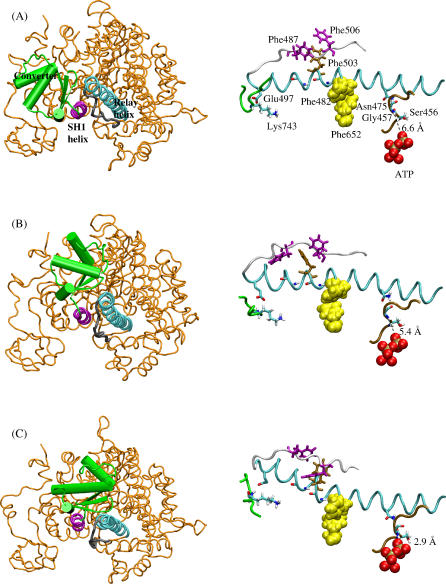
Three Snapshots (at 0.0 ps, 630.0 ps, and 1270.0 ps) from One of the Three TMD Simulations, in the Same Format as Figure 2 in [31] for Comparison (Left) The converter domain rotation, viewed from the C-terminus of the relay helix and down the axis of the SH1 helix, whose axis is similar to the converter-rotation axis. (Right) The proposed coupling between the small motion of the Switch II with the large translation of the helix C-terminus along the relay helix.

**Figure 4:**
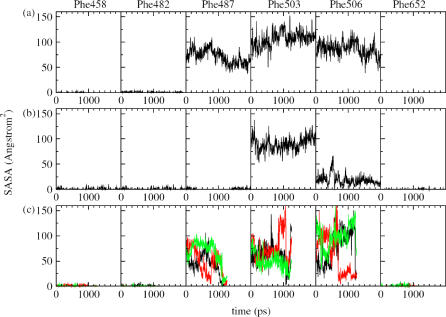
Solvent-Accessible Surface Area for a Number of Key Hydrophobic Residues in Equilibrium and TMD Simulations The Lee-Richards–type SASA [77] for Phe458, Phe482, Phe487, Phe503, Phe506, and Phe652 in the equilibrium simulations 1FMW (A) and 1VOM (B), and in the three TMD simulations (C) where different colors represent three different TMD trajectories. A water probe with radius 1.4 Å and the Nina-Roux set of atomic radii [74,75] are used. The GBSW model is used in both equilibrium and TMD simulations.

Key geometrical properties along the TMD trajectories (Figure 2) and three snapshots (at 0.0 ps, 630.0 ps, and 1270.0 ps) taken from one of the three TMD simulations (Figure 3) are shown to make a direct comparison with the MEP results reported in [31]. In contrast to the MEP results [31], little change is observed for the local structural rearrangement around the active site for the first 600 ps of the TMD trajectories, and most of the changes in this period come from movements in the C-terminus of the relay helix and the converter domain (see Figure 2). In this first phase (Figure 3A–3B), the converter domain rotates by ~70°–80° while the hydrogen bond between Gly457 and ATP is not formed. However, the salt bridge between Arg238–Glu459 quickly forms at the beginning of the transition, similar to what has been observed in the MEP study [31]. The hydrogen bond between the carbonyl of Ser456 and the side chain of Asn475, which has been regarded as the crucial interaction that couples the SwII and the relay helix in the MEP study [31], is persistent, once formed, during the entire trajectory in two of the three TMD simulations (38% and 62% occupancy, respectively) but not in the other TMD simulation. Other important hydrogen bonds between the relay helix and Switch II that form during this phase of the transition include Asn472 O^δ1^–Glu459 HN, Asn472 H^δ21^–Glu459 O, and Gln468 H^ɛ22^–Glu459 O^ɛ2^, which contribute together during the powerstroke recovery in stabilizing the N-terminal part of the relay helix. Residues Asn472, Asn475, His572, Tyr573, and Ala574 form a hydrophobic core for Phe458 in Switch II. During the transition, the swing and rotation of Phe458 are accompanied by the changes of these residues with Phe458 buried in the entire process (Figure 4), which helps to stabilize the relay helix. Accompanied by the translation of the C-terminal of relay helix, the relay helix becomes straighter (Figure 2B; note that in the 1FMW structure, the relay helix is not ideally straight), as seen in the MEP study [31].

In the second phase (Figure 3B and 3C), both the kink in the relay helix and the key hydrogen bond between Gly457 and ATP are formed (Figure 2B and 2C). The “unwinding” of the relay helix, characterized by the broken hydrogen bond between Met486 and Glu490, happens much later (at ~900 ps, see Figure 2C) in current TMD simulations, compared with the MEP study [31]. There are several aromatic residues located halfway in/around the relay helix (i.e., Phe482, Phe487, Phe503, Phe506, and Phe652), and their packing contributes to the formation of the kink in the relay helix. The Phe482 and Phe652 are entirely buried, arranged in T-shape stacking, during the entire transition (Figure 4). It has been proposed in the MEP study that this packing provides a pivoting point for the unbending of the relay helix [31]. The Phe487 reorients with the movement of relay helix in the first phase and finally in parallel packs against Phe506 in the second phase with their solvent accessible surface areas (SASAs) decreasing from ~70 Å^2^ and ~85 Å^2^ to ~7 Å^2^ and ~15 Å^2^, respectively.

During the transition, the converter domain makes contact with the relay helix via polar (hydrogen bonds and salt bridge) as well as hydrophobic interactions. The hydrophobic contacts that are present in all three TMD simulations include Met486–Ile687, Ile499–Phe692, Ile499–Arg738, Ile499–Phe745, Phe503–Lys690, and Phe506-Ile687, besides the hydrophobic core formed halfway in/around the relay helix (see Table A4 in Protocol S1). As transition progresses toward the pre-powerstroke state, a network of polar interactions form between highly conserved acidic residues (Glu490, Glu493, and Glu497) in the C-terminal of the relay helix and positively charged residues (Arg695, Lys743, and Arg738) within the SH1 helix and converter, which are also highly conserved. For example, Arg695 switches its hydrogen bond acceptor from Glu490 to Glu493. It is worth noting that some of the polar interactions, such as the hydrogen bond between Asn483 and Glu683, are not present in the equilibrium simulation of either end-states but only form during the transition to help induce or/and stabilize the kink formation in the relay helix.

In short, different types of interactions (polar versus hydrophobic) along the relay helix play an important role during the recovery stroke. Around halfway in the relay helix, the hydrophobic cluster provides stabilization to the kink of the relay helix, while at the joint between the relay helix and the relay loop region, strong polar interactions facilitate cooperative changes in the relay helix, the SH1 helix, and the converter domain.

### Trp501 in the Transition

**Figure 5:**
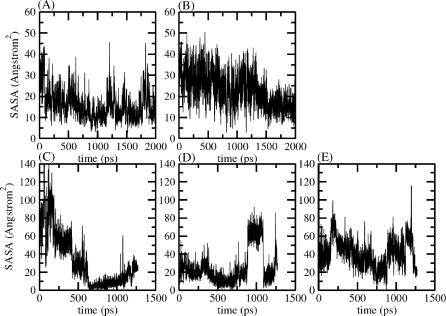
Solvent-Accessible Surface Area for Trp501 in Equilibrium and TMD Simulations The Lee-Richards–type SASA [77] for Trp501 in the equilibrium simulations 1FMW (A) and 1VOM (B) and in the three TMD simulations (C–E). A water probe with radius 1.4 Å and the Nina-Roux set of atomic radii [74,75] are used. The GBSW model is used in both equilibrium and TMD simulations.

During the TMD trajectories, the *main chain* of Trp501 has only moderate fluctuation similar to that observed in the equilibrium GBSW (generalized Born with a simple switching) simulation of 1VOM (unpublished data). In the equilibrium simulations (Figure 5A–5B), the SASA of Trp501 is about 15.8 ± 6.7 Å^2^ for 1FMW and 22.7 ± 8.1 Å^2^ for 1VOM. In the TMD simulations, however, diverse behaviors are observed (Figure 5C–5E), in contrast to the observation in the MEP study [31]: Trp501 can be fully exposed to solvent or almost fully buried by other groups during the transition; at the two endpoints of the TMD simulations, however, Trp501 does have a very similar SASA as that observed in the equilibrium simulations.

**Figure 6:**
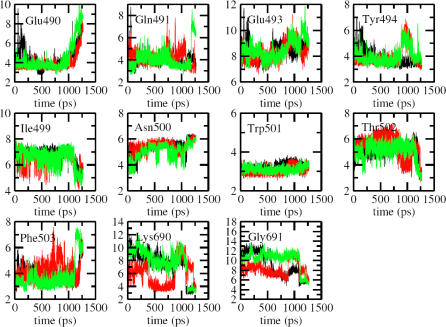
The Minimal Distances (in Å) in the TMD Simulations between Trp501 with Sidechains and Backbone Carbonyl Group Atoms That May Contribute to the ATP-Induced (De-)Quenching of Trp501 Fluorescence These groups include Glu490, Gln491, Glu493, Tyr494, Ile499 (backbone carbonyl), Asn500, Trp501 (backbone carbonyl), Thr502 (backbone carbonyl), Phe503 (backbone carbonyl), Lys690 (including backbone carbonyl), and Gly691 (backbone carbonyl) as suggested by Málnási-Csizmadia et al*.* [40]. The different colors represent three TMD simulations.

We also monitor the distance between Trp501 side chain and other nearby groups that may contribute to fluorescence quenching as suggested by Málnási-Csizmadia et al. [40]. As shown in Figure 6, all distances that show a decrease going from 1FMW to 1VOM involve backbone carbonyl groups (Ile499, Thr502, Lys690, and Gly691). By contrast, the distances from Glu490 and Phe503 to Trp501 increase systematically during the late stage of the transition.

### Hinge Analysis and SCA

To gain further insights into the structural features of the motor domain that are important to the coupling between the active site and other essential structural motifs, normal mode analysis [32,33] is carried out, which allows a systematic probe of structural flexibility and the identification of hinge residues that are presumably critical for flexibility. As a complementary approach, the SCA [14,34] is also used to search for closely coupled residues from the informatics perspective.

### Structural Flexibility and Motional Correlation

**Figure 7:**
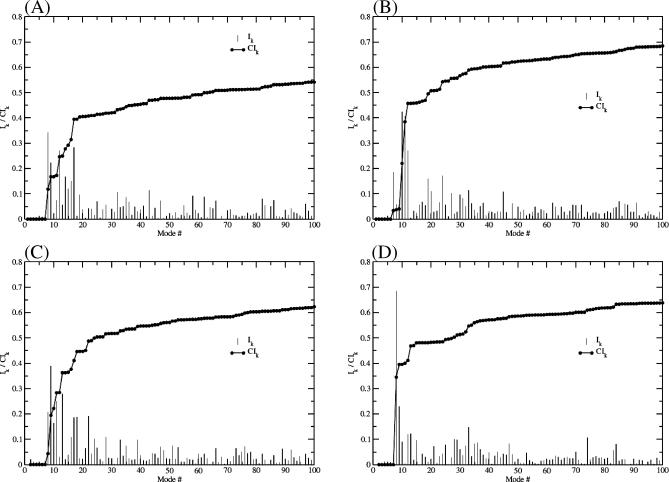
Involvement Coefficient (*I_k_*) and Cumulative Involvement Coefficient (*CI_k_*) from the Normal Mode Calculations (Equations 1 and 2) (A) The *I_k_*/*CI_k_* are calculated based on the normal modes obtained for 1FMW and mapped onto 1VOM. (B) The *I_k_*/*CI_k_* are calculated based on the normal modes obtained for 1VOM and mapped onto 1Q5G. (C) The *I_k_*/*CI_k_* are calculated based on the normal modes obtained for 1Q5G and mapped onto 1FMW. (D) The *I_k_*/*CI_k_* are calculated based on the normal modes obtained for 1VOM and mapped onto 1FMW.

Overall, the normal mode results are consistent with the previous analysis [25] using the block normal mode approach [41,42], despite the fact that slightly different procedures were used to generate missing residues in the X-ray structures. The structural fluctuations calculated based on the normal modes are also in semiquantitative agreement with those from the final 1 ns out of ~3-ns equilibrium MD simulations for the 1FMW and 1VOM states using the GBSW model (unpublished data); both states show considerable flexibility in the C-terminal converter domain, the N-terminus, and a loop region in the upper 50 K domain (one of the two actin-binding clefts). The correlation between the structural flexibility, as reflected by the low-frequency modes, and the conformational transition is quantitatively illustrated by the involvement coefficients (Equations 1 and 2). Similar to the previous analysis of the 1FMW → 1VOM transition [25] and a study on the scallop myosin [43], only a small number of modes have significant involvement coefficients for all sets of transitions analyzed here (Tables B1–B4 in Protocol S1). Moreover, for all sets of transitions, the cumulative coefficients show (Figure 7) that more than 40% of the total structural change can be captured by motions associated with the ten lowest-frequency intramolecular modes.

**Figure 8:**
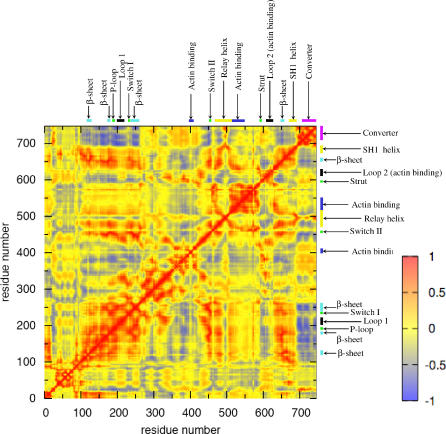
Covariance Matrix (Correlation Map) of the Cα Atoms in 1FMW Based on the Lowest 14 Normal Modes (Equations 4 and 5) Calculations using all calculated (100) normal modes give qualitatively similar results, although the magnitude of long-range correlation is smaller. The covariance matrices for the other two structures (1VOM and 1Q5G) are also qualitatively similar and therefore not shown.

Regarding the covariance matrix between all residue pairs, similar correlation patterns are observed for 1FMW, 1VOM, and 1Q5G, and therefore only the correlation map for 1FMW is shown in Figure 8. A number of features in the correlation maps are immediately evident. Residues within each structural element are found to exhibit cooperative motions, which is consistent with the fact that the three states are related mainly through motions of relatively rigid domains. The motions of the P-loop (179–186), Switch I (233–238), and Switch II (454–459) are highly correlated with each other. Moreover, their motions are correlated with the relay helix, the relay loop (466–518), and the SH1 helix (669–690). It is worth noting that such high correlations cannot be observed in the equilibrium GBSW simulations of 1FMW and 1VOM (unpublished data), which is likely due to the limited nanosecond time scale of the MD simulations. A negative correlation is found between the active-site residues and the strut motif (590–593). The converter domain has significant anticorrelation with the central ß sheets and loop 1. Finally, by examining the covariance in detail, it is found that the correlations between Phe482 and Gly680, and Ile499 and Phe692, are among the highest around the kink region in the relay helix, which once again highlights the importance of interactions between these residue pairs in maintaining the mechanochemical coupling.

### Hinge-Residue Analysis

**Figure 9:**
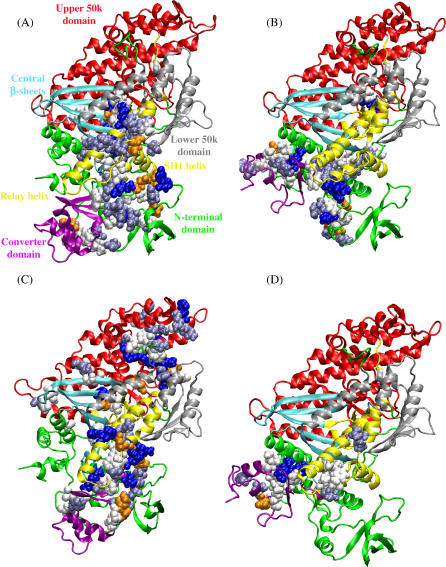
The Hinge Residues (Shown in the van der Waals Form) Identified by DynDom [84,85] Based on Results of Normal Mode Analysis See Table 2 and in Protocol S1 Tables B1–B4 and B6. Upper 50-kD domain, lower 50-kD domain, converter domain, and central β sheet are shown in red, silver, purple, and cyan, respectively. Strut loop, relay helix, P-loop, Switch I and Switch II, and SH1 helix are shown in yellow. The rest of the protein is shown in green. The hinge residues are colored based on residue type—blue, basic; orange, acidic; slate blue, polar; white, nonpolar. The four figures are based on modes with large involvement coefficients for different transitions: (A) 1FMW → 1VOM, (B) 1VOM → 1Q5G, (C) 1Q5G → 1FMW, (D) 1VOM → 1FMW.

Once the modes with large involvement coefficients are identified, it is of interest to locate the hinge residues in those modes, since it is sensible that these hinges dictate the structural flexibility relevant to function. The lists of bending and hinge residues identified for all modes with involvement coefficients larger than 0.10 are summarized in Protocol S1B for all sets of transitions considered, and the spatial distribution of these hinge residues is summarized in both Table 2 and Figure 9.

**Table 2:**
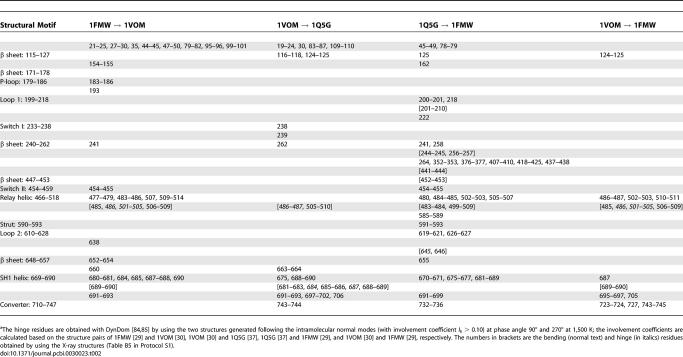
Spatial Distribution of Hinge Residues Listed in Tables B1–B5 in Protocol S1 ^a^

It is interesting to note that the distribution of the bending/hinge residues depends on both the state and the nature of the conformational transition because motions along different modes are relevant in different transitions. For example, Asp454 and Ile455 function as “pivoting residues” for the open/close movement of Switch II during the transition from 1FMW to 1VOM and from 1Q5G to 1FMW [44]; however, they do not play a major role in the transition from 1VOM to 1Q5G. In 1FMW (Figure 9A), for which we only consider the transition toward 1VOM, the hinge residues in the six dominant modes occur mainly in the N-terminal domain, the P-loop, and Switch II, as well as in the relay helix. In 1VOM, when the transition toward 1FMW is considered (Figure 9D), the bending/hinge residues are mainly found in the relay helix, SH1 helix, and converter domain; two hinges (Val124, Ala125) are also found in the central ß sheet region. When the transition toward the near-rigor state (1Q5G) is considered (Figure 9B), however, more hinge residues are found in the N-terminal region and the central ß sheet, while no hinges are found in the relay helix. The transition from 1Q5G to 1FMW seems most complex and involves a large number of low-frequency modes and correspondingly a large number of hinge residues distributed in all-important structural motifs (see Table 2 and Figure 9C). Another interesting feature in 1Q5G is that there exist hinges near two loops in the actin-binding interface (Table 2), which have been postulated to be functionally important (see below) [36,45–49].

The hinge analysis based on important low-frequency normal modes and crystal structures for different functional states reveals a rich amount of information regarding candidate residues important for the conformational transitions between these states. Indeed, if dynamical domain decomposition and hinge analyses are done based on the X-ray structures alone, only a handful of hinge residues in the relay helix and near the converter domain can be located (Table B5 in Protocol S1B.

### Statistical Coupling Analysis

**Figure 10:**
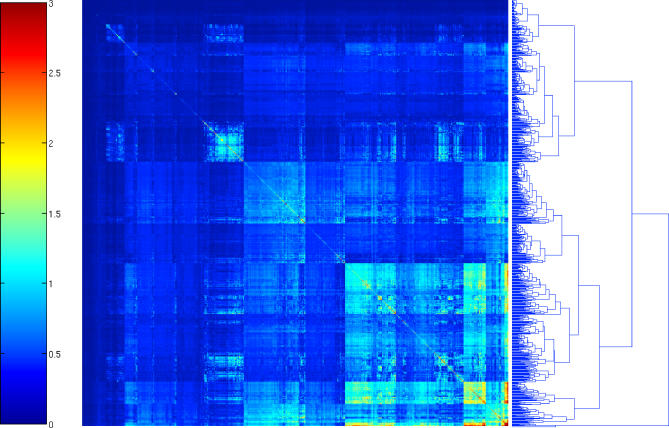
The Entire Matrix of Pair-Wise Coupling Energies of Myosin Motor Domain Residues Plotted with blue representing the lowest coupling energy (0 kT*) and red representing the highest coupling energy (3 kT*), respectively. kT* is an arbitrary energy unit. Both horizontal and vertical axes represent the residue number (1–761) of *Dictyostelium* myosin motor domain (following the clustering analysis).

In the entire coupling matrix obtained by SCA [14,34] (Figure 10), most regions have low coupling energies except for those on the bottom right (following the clustering analysis). The mean coupling energy (mean) and standard deviation (SD) of coupling energies between all residue pairs are 0.40 kT* and 0.32 kT*, respectively (kT* is an arbitrary energy unit). Following hierarchical 2-D clustering around the high-energy region in Figure 10, with low-energy regions (less than 1.36 kT*) discarded during each iteration, a group of 52 self-consistently coupled residues are obtained. The histogram of coupling energies between these residues (Figure B2B in Protocol S1) shows a mean value of 1.72 kT*, implying that these core residues are coupled to each other significantly.

**Figure 11:**
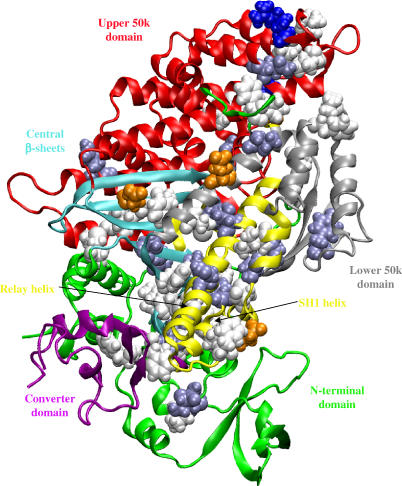
Mapping of the 52 Strongly Coupled Core Residues (in van der Waals Form) to the Structure of *Dictyostelium* Myosin Motor Domain (PDB Code 1VOM) The structure of the motor domain is displayed using the cartoon scheme with the same color scheme as Figure 9. The coupled residues are colored based on residue type: blue, basic; orange, acidic; slate blue, polar; white, nonpolar.

As shown in Table 3, these 52 significantly coupled residues distribute globally in the motor domain and occupy sites in the intersubdomain linkers such as Switch II, relay helix, SH1 helix, strut, and the central ß sheet, many of which have been speculated to play an important role in maintaining the mechanochemical coupling (see Discussion). Mapping the 52 coupled residues onto the 1VOM structure (Figure 11) shows more clearly that a cluster of coupled residues appear in the intersection of four subdomains and extends from the upper 50-kD domain, nucleotide-binding pocket, and lower 50-kD domain to the converter domain. Most of them are located in the key regions including intersubdomain linkers (yellow), the central ß sheet (cyan), and the actin-binding interface (Figure 11).

**Table 3:**
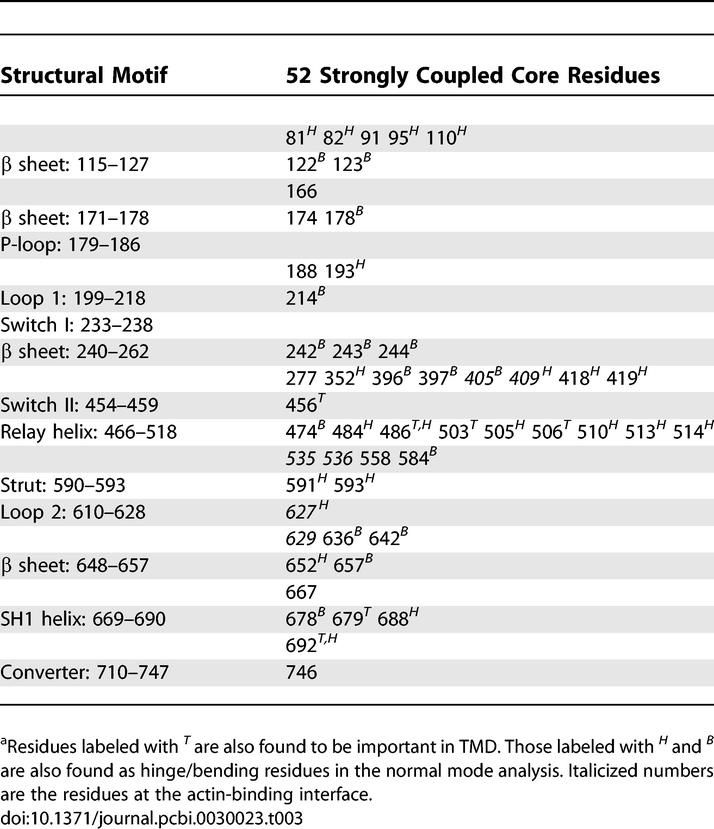
Spatial Distribution of 52 Strongly Coupled Core Residues Obtained Using the SCA [14,34]^a^

## Discussion

In the following, we first make connection between the current simulations and a previous computational study regarding the mechanism for the recovery stroke [31] and Trp501 fluorescence experiments [35,38–40]. Next, we discuss the residues that have been identified as important to the mechanochemical coupling by the combination of several computational methods.

### Relation between the TMD Simulations to the Previous MEP Analysis

The TMD analysis provides an approximate description of the actual transition process and is also useful for identifying key residues (see discussions below). It is worth noting that the TMD results deviate substantially from the MEP results of Fischer and co-workers [31]. The MEP study produced a highly ordered transition path that initiates from the active site and propagates sequentially through the relay helix to the converter domain. The TMD results, however, indicate that most rotation of the converter domain occurs in the first stage of the transition, while structural changes in the relay helix and complete closure of the active site occur at a later stage to stabilize the converter conformation via a series of hydrogen-bonding interactions as well as hydrophobic contacts (see Tables A1–A4 in Protocol S1A). For example, the “unwinding” of the relay helix happens much later in the TMD simulations compared with the MEP description [31]. In contrast to the MEP result, which suggests that the kink and unwinding in the relay helix are induced by the SwII closure via a single hydrogen-bonding interaction involving Asn475, the TMD simulations tend to suggest that the converter rotation, via strong polar interactions to the relay helix and the relay loop, induces the formation of the hydrophobic cluster halfway in the relay helix as well as some polar interactions (e.g., Asn483–Glu683) to produce the kink in the relay helix. The interactions between the Sw II and the relay helix stabilize, in return, the new conformation of the relay helix.

Which description is closer to reality is debatable because both methods have limitations. The MEP approach does not include the effect of thermal fluctuation and therefore tends to produce highly ordered sequences of events; moreover, the study of Fischer et al. [31] only examined one path, which may not be the most representative. The diversity between different paths is illustrated by the behavior of the crucial Asn475, which is found to maintain its hydrogen bond with Ser456 in two TMD trajectories but not in the third; another example concerns the widely used reporter in fluorescent experiments, Trp501, which exhibits much more complex behavior in the TMD than in the MEP results. The TMD approach includes thermal fluctuations, yet the transition path is subject to artifacts introduced in the form of the constraint; it has been argued that the enforced monotonic decrease of the holonomic RMSD constraint would artificially bias large-scale conformational rearrangements in the early stage of transition. Although this is consistent with the finding here that converter rotation occurs in the first phase of the TMD trajectories (Figures 2 and 3), the fact that the converter domain is flexible, as indicated by normal mode analysis [25], experimental FRET measurements [50], and electron microscopic study of Trinick and co-workers [51], suggests that this is not physically unreasonable. The existence of decoupling mutants in which ATP hydrolysis occurs with normal rate but in which motility gets abolished seems to support the idea that the active-site closure precedes the converter domain rotation [12,13]. However, this can also be understood as active-site closure and a large degree of converter rotation being able to occur independently, but key residues are required to couple the two transitions. In fact, many recent studies of allosteric systems tend to support the mechanism in which large-scale motions occur (at least to a significant degree) prior to activation, and the role of the activation event is to stabilize, rather than induce, the activate conformation [5,52]. Evidently, the interplay between motions along soft, collective modes, and local, sequential structural rearrangements in allosteric systems such as molecular motors remains to be thoroughly explored [53,54]. Experimentally, powerful tests would include X-ray structures for mutants or inhibitor complexes that might trap the system in a transitional structural state, as has been done for ras-p21 [55] and CheY [56,57]; in these two examples, the X-ray structures in fact provided strong support for TMD-type simulations of these systems.

The encouraging aspect, on the other hand, is that both TMD and MEP studies point to the importance of a consistent set of hydrophobic interactions (e.g., between Phe482, Phe487, Phe503, Phe506, and Phe652) and hydrogen-bonding interactions (e.g., Glu497–Lys743) between the relay helix, active-site, and the converter domain (see below for detailed discussions). A richer set of interactions has been identified in the current analysis, due partly to the fact that a more diverse set of configurations are accessible in the TMD simulations. Therefore, the TMD- and MEP-based studies are complementary to each other in the study of highly complex structural transitions in macromolecules.

### Relation to Previous Fluorescence Experiments Employing Trp501

Since the fluorescence of Trp501 has been used extensively in experiments as an observable to monitor the behavior of the motor domain during the functional cycle [38–40], it is important to establish what factors modulate this observable and therefore what information can be inferred from its change [35]. The molecular dynamics simulations carried out here have provided an opportunity for doing so.

As discussed in Results, the mainchain of Trp501 has only moderate fluctuation in both TMD and equilibrium simulations of the motor domain. This is consistent with what has been suggested by Shih and Spudich [58] and confirmed by Málnási-Csizmadia et al. [40]: the relay/converter interface remains intact in the actin-bound state of myosin and throughout the entire actin-activated ATPase cycle; the ATP-induced fluorescence enhancement associated with the recovery transition is *not* because the relay loop goes from a disordered to an ordered state and internalizes Trp501, thus protecting it from solvent (H_2_O) quenching.

The diverse behavior of the Trp501 SASA observed in the TMD simulations (Figure 5C–5E), which was difficult to sample in the MEP study [31], further supports the proposal that the accessibility of water should not contribute much to ATP-induced fluorescence enhancement [40]. Instead, the variations in the distances between Trp501 sidechain and nearby groups (Figure 6) suggest that during the 1FMW → 1VOM transition, the fluorescence enhancement of Trp501 might be caused by the de-quenching effect due to the displacement of the Glu490 side chain and the Phe503 carbonyl, basing on the systematic increase in their distances to Trp501. These observed structural changes are consistent with the conclusion drawn from fluorescence experiment that Trp501 has at least three different microenvironments [40], which highlights the complexity inherent in the interpretation of such experiments.

### Residues Important to Mechanochemical Coupling

In the current study, the basic framework of identifying residues important for mechanochemical coupling involves the analysis of residues involved in altered interactions during the TMD simulations, the hinge residues in functionally relevant normal modes (as measured by the involvement coefficients), and the SCA. The hinge-residue analysis addresses the role of collective motions (flexibilities), while the interaction analysis addresses the role of local interactions; the SCA, on the other hand, is based on sequence information only. Therefore, the three approaches are likely to be complementary to each other. Other methods for addressing the collective [15,16] and local [59] motions in allostery are available and may be used; the vital point here is to advocate addressing both types of contributions [54]; most previous studies focused on only one of them.

The TMD results reveal that both hydrophobic and polar interactions (see Protocol S1A) play a role in the recovery stroke. For example, in the first phase of the transition, Phe458 in Switch II swings and rotates while remaining buried with hydrophobic interactions with the nearby residues (Figure 4), which helps to stabilize the relay helix. This observation is consistent with the mutation analysis by Sasaki et al*.* [44], who showed that F458A loses the actin-activated ATPase activity without loss of the basal ATPase activity. During the second phase of the transition (Figure 3), a set of aromatic residues (Phe482, Phe487, Phe503, Phe506, and Phe652) are observed to pack to facilitate the formation of the kink in the relay helix, which has also been observed in the previous MEP [31]. This result explains the mutagenesis experiments showing that the mutation F487A or F506G uncouples the ATPase and the lever arm motion [60]. Finally, at the converter/relay interface, a set of hydrophobic interactions are also observed to form during the transition, and the implicated residues include Met486, I499, Phe503, Phe506, Ile687, Lys690, Phe692, and Arg738. It has been shown experimentally by Shih and Spudich that the interface between the relay helix and converter remains intact throughout the actomyosin ATPase cycle [58], which emphasized the importance of these hydrophobic contacts. Indeed, mutagenesis experiments have shown that I499A and F692A completely abolish the motor function of myosin II in vivo and in vitro, although the ATPase activity is not altered [13]. Regarding polar interactions, a few residues identified include Gln468, Asn472, and Asn475 at the interface between Switch II and the relay helix, and Glu493, Arg695, Lys743, and Arg738 at the interface between the relay helix and the converter. Sequence alignment shows that all these polar residues except Arg738 are highly conserved (>80%) among myosin family members, which highlights the importance of these three residues.

Normal mode analyses clearly reveal strong motional correlation (Figure 8) among active-site motifs (Switch I, Switch II, and P-loop) and between these motifs with the relay helix, relay loop, and SH1. These correlations indicate that structural motifs of the motor domain are spatially distributed such that conformational transitions can be propagated over a long distance. Regarding the communication between the active-site and the actin-binding interface, for example, the cross-correlation analysis captures the negative correlation between the strut motif (590–593) and the active site, which supports the hypothesized role of the Switch I close/open in modulating the open/close of the actin-binding clefts [15,23,37,61,62].

Among all the hinges identified, a small but significant fraction (39, see Table B6 in Protocol S1) are highly conserved (>80% across all species), which supports their functional importance. Among the rest, 21 are overlapped, with the result of the SCA and the others having varying degrees of conservation that range from ~30% to ~80% (Figure B1 in Protocol S1). Among the latter subset, most of the hinge residues occur in regions close to highly conserved or SCA-identified residues, with the only exception occurring in the N-terminal domain.

Overall, despite the fact that the hinge analysis and SCA are based on very different principles, with the hinge analysis being more sensitive to what structural transition is being analyzed, many common residues emerge (see Table 3). The most notable lists include regions near Ser456 in Switch II, a number of residues in the relay helix (484, 486, 503, 505, 506, 510, 513, 514), 591 and 593 in the strut, and several residues in the central ß sheet (~123, ~241, 652, 654). Also highlighted in both analyses are two loop regions that have been implicated in previous experimental studies. Loop 1 (199–218), a flexible surface loop in the myosin motor domain, has been proposed based on structural studies to be responsible for the kinetic tuning of product release following ATP hydrolysis [36]. Biochemical studies have shown that loop 1 affects the affinity of actin–myosin for ADP, motility, and the *V_max_* of the actin-activated ATPase activity, possibly through P*_i_* release [21,63–65]. Loop 2 (610–628) has been shown to play an important role in regulating nucleotide release and actin affinity [45–49]. Finally, eight residues in the strut and the actin-binding interface (Table 3) have been shown by SCA to be coupled with other residues spread over all functionally important structural motifs.

The fact that a large fraction of the SCA core residues are either hinge/bending residues identified in the normal mode analysis or found to engage in important interactions in TMD simulations (Table 3) highlights the value of combining SCA and physically motivated analyses, because the latter assign possible functional roles to the identified core residues. Indeed, since the SCA algorithm works with sequence information only, the identified residues are not guaranteed to involve allostery and might instead play a role in, for example, cooperative folding. On the other hand, residues revealed in the SCA analysis are particularly interesting because, by definition, these residues are at most moderately conserved (~40%–80% in the current analysis) and therefore may not have caught the attention of mutagenesis studies. Nevertheless, several residues in the list have been analyzed. Spudich and co-workers [66] demonstrated that the mutant S456L in Switch II partially uncoupled ATP hydrolysis and myosin motility, resulting in a rather small step size only one-tenth that of the wild-type. The F692A mutation in the SH1 helix was found to abolish the in vitro and in vivo motor functions without significantly affecting the myosin ATPase activity [13]. The T474P mutant also showed uncoupling of actin-activated ATP hydrolysis from in vitro motility to some extent [67]. Finally, several mutations of the reactive cysteine residue Cys678 in the SH2 helix were found to retain similar actin-activated ATPase hydrolysis activity but with reduced sliding velocity [68]. Given these reported cases, it seems that SCA is indeed capable of identifying key residues for long-range communication in biomolecules, and we strongly encourage further experimental tests of the other identified sites.

### Conclusions

To fully understand the mechanism of mechanochemical coupling in myosin, it is important to reveal residues and interactions that dictate the coordination among ATP hydrolysis, active-site open/close, converter rotation, and changes in the actin-binding site. This is a challenging problem due to its allosteric nature and has been undertaken using a combination of computational approaches in the present systematic analysis. The normal mode analysis addresses the role of collective motions (flexibilities), while the TMD simulations focus on the role of specific local interactions during the transition, and the SCA examines the problem by analyzing sequence information during evolution. The complementarities between the results have produced valuable information regarding residues that play a role in propagating “signals” between the active site and distant functional sites. Mutation of some of these residues have been shown experimentally to give rise to “decoupling mutants” in which the ATPase activity of the motor domain is largely unaffected while the motor function is compromised or even abolished. Such validations urge us to encourage future experimental analysis of other residues identified in the current work as important regulatory elements of the mechanochemical coupling, especially those core residues identified in the SCA since they tend to be at most moderately conserved despite functional significance.

The simulations also have allowed us to make connections to previous computational and experimental studies in a critical manner. The behavior of the widely used reporter residue, Trp501, in the simulations confirms the concern that its fluorescence does not simply reflect the relay loop conformation or active-site open/close but depends subtly on its microenvironment. The findings in the TMD and a previous MEP analysis [31] of the recovery stroke have been compared and analyzed, which emphasized the differences and complementarity of the two approaches. The MEP approach neglects the effect of temperature and tends to produce highly ordered sequences of events; the TMD approach is subject to the approximate nature of the applied constraints, although the observation of large-domain motions in the early stage of the trajectory is consistent with the intrinsic structural flexibility of the system, a fundamental feature that has been increasingly appreciated for many motor and allosteric systems [5,6,69]. The encouraging aspect is that a similar set of interactions can be identified with both approaches as potentially important in mediating long-range structural transitions, although TMD tends to reveal a richer set of interactions due to the more extensive configuration sampling at the finite temperature.

In short, by judiciously combining a range of computational techniques with different approximations and strength, we have established a clearer mechanistic picture regarding the energetic and structural features that dictate the coordination between ATP chemistry and conformational changes; i.e., the mechanochemical coupling, in the myosin motor domain. The modulation of structural flexibility at both the local and domain scales with strategically placed “hotspot” residues and phosphate chemistry is likely the general feature for mechanochemical coupling in many molecular motors. Finally, the fundamental strategies of examining both collective and local changes and combining physically motivated methods and informatics-driven techniques are expected to be valuable to the study of other molecular motors and allosteric systems in general.

## Materials and Methods

### Equilibrium and TMD simulations with implicit solvent model.

Due to the intrinsic, long time scale associated with the recovery stroke, TMD [27,28] simulations are performed to enforce the transition from the post-rigor state (1FMW with ATP) to the pre-powerstroke state (1VOM with ATP). As reference, equilibrium simulations are also carried out for the two end states. In those simulations, the protein atoms and the Mg^2+^ ion in the active site are represented by the all-atom CHARMM22 force field [70], and the four water molecules in the active site are treated with the TIP3P model [71]. Other water molecules observed in the X-ray structures and the bulk solvent are replaced by an implicit solvent model to reduce the computational cost. Specifically, the generalized Born model with a simple switching function (GBSW) is used [72]. With this setup, for different states (1FMW:ATP, 1VOM:ATP, and 1VOM:ADP·P*_i_*), the backbone RMSDs fluctuate around 3.2 Å after about 1-ns equilibration and 3-ns production run (unpublished data), which is reasonable considering the relatively large size of the system. The starting and target structures for the TMD simulations are chosen as the energy-minimized structures of 1FMW and 1VOM based on the X-ray data. All bonds involving hydrogen are constrained with the SHAKE algorithm [73], using a relative geometric tolerance of 10^−10^, and the time step is set to 1 fs. A 16-Å cutoff is applied for nonbonded interactions with a switch function between 14 Å and 16 Å, and the nonbond list is updated with a heuristic algorithm. For the GBSW calculations, a smoothing length of 0.3 Å at the dielectric boundary with 24 radial integration points up to 20 Å and 38 angular integration points are used. The nonpolar solvation free energy is computed using a surface tension coefficient of 0.03 kcal mol^−1^ Å^−2^, as used in the original development of GBSW [72]. Since the GBSW model was parameterized to closely to mimic the solvation energies from the finite-difference Poisson method, the optimized radii for the latter method [74,75] are adopted to define the solvent–solute dielectric boundary. The system is weakly coupled to a temperature bath at 300.0 K with a relaxation time of 5 ps [76]. The system center of mass motion is removed every 1,000 time steps to prevent the buildup of center of mass velocity. In the TMD setup, the fitting set is chosen to include all backbone atoms while the holonomic constraint is applied to all the nonhydrogen protein atoms. Every step, the RMSD to the target structure is decreased by 5 × 10^−6^ Å. For the transition from the post-rigor state to the pre-powerstroke state, three different random seeds are used to assign the initial velocities to produce three independent TMD simulations. Approximately 1.3-ns simulation is needed for each transition to complete after equilibration.

To explore factors that might modulate the Trp501 fluorescence, we monitor the SASA for Trp501 in the TMD simulations as well as in the equilibrium simulations [77] with a water probe of 1.4 Å and the Nina-Roux set of atomic radii [74,75].

### Normal mode analysis.

To determine the structural flexibility of the myosin motor domain, normal mode analysis is performed on several structures including the post-rigor (1FMW), pre-powerstroke (1VOM), and near-rigor (1Q5G) states. The calculations are done with an implicit-solvent model, EEF1 [78]; accordingly, the Param19 polar hydrogen force field [79] is used. All models are minimized starting with the corresponding X-ray structure using the adopted basis Newton-Raphson method. All minimizations are carried out in an iterative fashion with a gradually decreasing harmonic restraining potential, which helps reduce unfavorable steric contacts without perturbing the structure significantly. The minimized structures have root-mean-square gradient less than 0.001 kcal mol^−1^ Å^−1^.

All normal mode calculations are carried out using the standard procedure implemented in CHARMM [80]. Only the first 100 lowest modes are calculated because they are expected to be the most important for the flexibility of the system and collective conformational change. The results of the normal-mode analysis are analyzed in terms of the root-mean-square atomic fluctuations (RMSF, 〈(Δ*r*)^2^〉^½^, unpublished data) and involvement coefficient (*I_k_* [81]); the latter is defined as

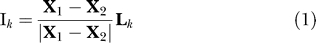
where **X_1_ − X_2_** is the displacement vector between two conformations (**X_1_**, **X_2_**) and **L_k_** is the *k*-th eigenvector. A large involvement coefficient indicates that the motion along this normal mode is highly relevant to the structural transition being examined between the two conformations. A related quantity that indicates the weight of a set of normal modes in a conformational transition is the cumulative involvement coefficients,

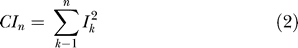



Based on the involvement coefficients, one can also estimate the effect of moving along *n* modes on reducing the RMSD between the two conformations:

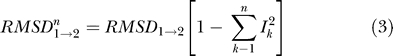
where *RMSD*_1→2_ is the total RMSD between the two conformations [69]. This quantity indicates how close the two conformers can be when *n* modes are followed, though the actual motion would depend on the energies in each of the modes.

Another quantity of interest is the covariance matrix or correlation map, which defines the motional correlation between a residue pair and is computed as follows [82]

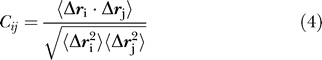
where

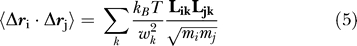



The results shown in Figure 8 include the 14 lowest-frequency modes (excluding overall translation/rotation modes); this number of modes is chosen because the cumulative involvement coefficients start to level off for all transitions considered here (see Figure 7), and including all calculated 100 modes, which is theoretically more sound [83], gives qualitatively similar results.

To further identify residues that dictate functionally important flexibility, hinge residues are analyzed for modes with large involvement coefficients (*I_k_* > 0.10) using the DynDom package [84,85]. Specifically, for each normal mode analyzed, two structures are generated by following the corresponding eigenvector with phase angles of 90° and 270°, respectively, at 1,500 K. These structures are then used as the two input structures for DynDom to decompose the structure into dynamical domains (to be distinguished from the structural domains identified based on static structure) and to identify hinge residues associated with the relative displacements of these dynamical domains. The minimum domain size is set to 20 residues, and the cutoff for the definition of effective hinge axis and mechanical hinge is set to 5.5 Å.

### Statistical coupling analysis.

The SCA approach developed by Ranganathan and co-workers [14,34] has been used as an alternative avenue for identifying residues that mediate the coupling between the active site and distant regions in the motor domain, such as the converter domain and the actin-binding interface.

We chose the N-terminal sequence (1–761 residues) of the *Dictyostelium* myosin II motor domain as a probe to search the NCBI refseq database [86] for myosin motor domain family members. In total, 709 sequences are collected using PSI-Blast [87] with an e-score ≤ 0.001. Multiple-sequence alignment is carried out using the program MUSCLE [88], and SCA is performed with the improved feature capable of calculating the coupling energies between all sites. Hierarchical 2-D clustering of the coupling matrix is applied until a group of self-consistently coupled residues that couple to each other with high energies is obtained. All SCA plots are generated using MATLAB 7.0 (Mathworks, http://www.mathworks.com).

## Supporting Information

Protocol S1 Mechanochemical Coupling in the Myosin Motor Domain: Analysis of Critical Residues (A) Important interactions. (B) List of bending/hinge residues.
Found at DOI: 10.1371/journal.pcbi.0030023.sd001.

## Abbreviations

GBSW: generalized Born with a simple switching
SASA: solvent accessible surface area
SCA: statistical coupling analysis
TMD: targeted molecular dynamics
