# Supplementary material for: Mechanochemical Coupling in the Myosin Motor Domain. II. Analysis of Critical Residues
Source: PLoS Comput Biol. 2007 Feb 16;3(2):e23. doi: 10.1371/journal.pcbi.0030023 (PMC1800309; doi:10.1371/journal.pcbi.0030023)
Supplement: Supplementary file 1 [file pcbi.0030023.sd001.pdf]

Protocol S1: Mechanochemical coupling in the myosin motor domain.

II. Analysis of critical residues

A Important interactions

Table A.1
Hydrogen bond between the Switch II and Relay helix[a]

Hydrogen bond	1FMW[b]	1VOM[b]	TMD 1	TMD 2	TMD 3
HE22(Q468)-OE1(E459)	11/75	44/99	19	5	21
OD1(N472)-HN(E459)	90/66	99/86	87	32	80
HD21(N472)-O(E459)	20/85	1/88	20	29	31
HD21(N475)-O(S456)	7/93	99/99	38	62	16
OD1(N475)-HN(F458)	0/0	30/19	0	0	1

a. The occurences (in %) of the hydrogen bonds between the Switch II and relay helix. The hydrogen bonds are determined based on a geometric criterion: the hydrogen-acceptor distance is shorter than 2.5 Å and the donor-hydrogen-acceptor angle is larger than 135 °. b. The two numbers refer to the occupancies from the implicit solvent (GBSW) and explicit solvent simulations, respectively. The GBSW model is used for all TMD simulations.

Table A.2
Hydrophobic interactions between the Switch II and Relay helix[a]

Hydrophobic contact	1FMW[b]	1VOM[b]	TMD 1	TMD 2	TMD 3
I455-T474	87/45	26/39	49	56	25
I455-L478	46/87	69/55	73	50	45
S456-I471	27/31	92/88	44	62	30

a. The occurences (in %) of the hydrophobic contacts between the Switch II and relay helix. The hydrophobic contacts are determined based on a geometric criterion: the non-hydrogen sidechain atoms between two hydrophobic residues are shorter than 4.0 Å. The occurence refers to the largest probabilities among all the atomic pairs for the two residues. b. The two numbers refer to the occupancies from the implicit solvent (GBSW) and explicit solvent simulations, respectively. The GBSW model is used for all TMD simulations.

Table A.3
Hydrogen bond between the relay helix and SH1 helix and converter domain[a]

Hydrogen bond	1FMW	1VOM	TMD 1	TMD 2	TMD 3
HE21(Q479)-O(C678)			11	15	
HE21(Q479)-O(N679)	49				41
HE22(Q479)-O(N679)		55			
HD21(N483)-OE1(E683)				28	
HD21(N483)-OE2(E683)			11		25
OE1(E490)-HH12(R695)	41		96	78	61
OE1(E490)-HH21(R695)				11	
OE1(E490)-HH22(R695)	82		24	30	20
OE2(E490)-HH12(R695)	55		12	16	43
OE2(E490)-HH22(R695)	22		85	75	67
OE1(E493)-HE(R695)		89			
OE1(E493)-HH21(R695)		51			
OE1(E493)-HZ1(K743)			38	11	36
OE1(E493)-HZ2(K743)			23	13	17
OE1(E493)-HZ3(K743)			22	11	23
OE2(E493)-HH11(R695)			81	18	75
OE2(E493)-HZ1(K743)		20		12	
OE2(E493)-HZ2(K743)		31		21	
OE2(E493)-HZ3(K743)		17		14	
O(E493)-HH12(R738)					52
O(E493)-HH22(R738)					27
OH(Y494)-HN(G691)			14		
OH(Y494)-HN(F692)	49	98	44	62	52
OH(Y494)-HH21(R695)	72		21		
OE1(E497)-HN(I741)	99			96	90
OE1(E497)-HN(T742)	98	31	27	82	77
OE1(E497)-HG1(T742)		37	43		10
OE1(E497)-HN(K743)	98		12	29	66
OE1(E497)-HZ2(K743)		11			
OE1(E497)-HZ3(K743)		14			
OE2(E497)-HN(I741)		87	89		
OE2(E497)-HN(T742)	21	85	89	39	43
OE2(E497)-HG1(T742)	53			79	58
OE2(E497)-HN(K743)		98	49		
O(N500)-HH11(R738)		23			
O(N500)-HH22(R738)		28			
OG1(T502)-HH12(R747)		83			
OG1(T502)-HH22(R747)		31			
HG1(T502)-O(K690)			14		
O(F506)-HE(R686)					16
O(F506)-HH21(R686)					33
HN(G507)-OE1(E683)		34			31
HN(G507)-OE2(E683)		40		17	10
OD2(D509)-HH12(R686)			12		
OD2(D509)-HH22(R686)			45		

a. See Fig.A.1 caption for details.

Table A.4
Hydrophobic interactions between the relay helix and SH1 helix and converter domain[a]

Hydrophobic contact	1FMW	1VOM	TMD 1	TMD 2	TMD 3
M486-I687	35	53	42	36	10
M486-T688			29		
F487-I687		42			
K498-R738			17		24
K498-I741		13			
I499-R738	57	48	25	33	10
I499-F692	61		56	74	20
I499-F745	80		87	48	14
W501-I687	15	12		13	
W501-K690		38		65	
T502-F692					58
T502-R747	22				10
F503-F687					18
F503-K690	19		56	47	74
I504-K690		25			
F506-R686		40	41		
F506-I687	29	49	43	10	21
F506-K690	12			20	

a. See Fig.A.2 caption for details.

B List of Bending/Hinge Residues

Table B.1
Results of dynamical domain partition and bending residues of 1FMW for the normal modes with involvement coefficient (I_k) larger than 0.10 obtained by DynDom(84; 85)[a]

Mode[b]	I_k [c]	No. [d]	Range of domains [e]	Bending residues [f]
8	0.34	4	1: 3-21,37-48,79-146,151-154,481-506,638-645,653-657,661-691	
			2: 22-27,693-745	1-2: 20,*21-22*,23-26,*690-693*
			3: 31-36,49-78	1-3: 36-37,*48-49*,78-79; 2-3: *22-25*,26,*27*,28-34
			4: 147-150,155-480,507-637,646-652,658-660	1-4: 146-147,150-152,*154-155*,480-481,504-507,637,*638*,*652-653*,657-658,660-661
9	0.22	4	1: 30-80,96-98	2-1: *80-81*,82-86,*95-96*,98,*99-101*
			2: 3-21,81-95,101-214,242-256,441-454,478-486,507-509,512-513,644-687	
			3: 22-29,487-506,688-745	2-3: 16-23,*483-486*,487-494,506-507,*684-685*,686-688; 1-3: 28-33
			4: 215-241,257-440,455-477,510-511,514-643	2-4: 214-215,*241*,242,256-257,440-441,*454-455*,*477*,478,*509-514*,643-644
12	0.27	5	1: 22-27,490-492,688-745	
			2: 32-35,37-37,50-79	3-2: *35*,36-46,48,*49-50*,76-79,*80-81*,82-86; 1-2: 27,*28-30*,31-32
			3: 3-21,36,44-49,80-183,194-201,449-457,476-489,493-506,509-510,640-660,663-687	3-1: 20,*21-22*,489-501,*687-688*
			4: 458-467,472-474,507-508,511-581	3-4: 457-458,472-476,506-511
			5: 184-193,202-385,417-448,468-469,590-639,661-662	3-5: *183-184*,*193*,194,201-202,448-449,639-640,*660*,661-663; 4-5: 466-474,581-590
14	0.17	3	1: 3-27,45-47,82-95,100-456,479-507,591-627,635-745	
			2: 28-44,48-79,97-99	1-2: *27-28*,*44-45*,*47*,48,79,*80-82*,95-99,*100*
			3: 457-478,508-590,628-634	1-3: 456-457,*478-479*,*507*,508,590-591,625-628,634-635
16	0.17	2	1: 9-16,30-480,508-645,648-680	
			2: 27-29,481-507,646-647,681-745	1-2: 16-27,29-34,480-481,507-508,645-649,*680-681*
17	0.28	3	1: 3-18,39-48,87-90,100-131,143,154,156-157,176-185,654-677	
			2: 28-38,49-80,96-99,497-507,678-745	1-2: 17-34,38-39,48-49, *79*, 80-87,90-96,*99-100*,677-678
			3: 186-192,215-241,257-258,260-441,454-496,508-637	1-3: *185-186*,637-652,*653-654*

a. The involvement coefficients are calculated based on the structure of 1FMW and 1VOM. b. the mode number (including the first 6 overall translation/rotation normal modes); c. the involvement coefficient for the normal mode; d. the number of dynamical domains obtained by using the two structures generated following a specific mode with phase angle 90° and 270° at 1500 K; e. the residue range for each domain; f. the bending residue range, and the first index for the domain refers to the fixed one for structural alignment. Italics indicates that the bending region is a mechanical hinge, *i.e.* contains a residue with its Cα within 5.5 Å of the interdomain screw axis.

Table B.2

Results of dynamical domain partition and bending residues of 1VOM for the normal modes with involvement coefficient (I_k) larger than 0.10 obtained by DynDom(84; 85)^b

Mode^b	I_k ^{c}	No. ^{d}	Range of domains ^{e}	Bending residues ^{f}
7	0.18	2	1: 697-703,705-735	
			2: 3-696,704-704,736-745	1-2: 681-687,*688-693*,694-697,700-706,735-745
10	0.42	5	1: 3-29	
			2: 30-107,110-113,115-116,673-673,675-688	3-2: 107-117,672-674,*675*,688-689
				1-2: 29,*30*
			3: 108-109,114,117-305,307,312-347,424-588,616-672,674-674,689-702,726-745	
			4: 306-306,308-311,348-423,592-615	3-4: 304-312,347-355,423-424,588-592,613-616
			5: 703-725	3-5: 702-703,725-742,*743-744*
11	0.40	2	1: 3-90,94-110,112-112,125-488,504-690	
			2: 91-93,111,113-124,489-503,691-745	1-2: 90-91,93-94,110-113,*124-125*,488-489,502-505,684-692
12	0.27	2	1: 3-20,85-745	
			2: 21-84	1-2: 3-18,*19-24*,25-28,*83-87*
19	0.16	4	1: 3-17,110-116,121-128,131-177,180-211,237,239-261,431-458,476-502,639-662,693-697,732-745	
			2: 29-109,117-118,178-179,664-683	1-2: 15-29,*109-110*,*116-118*,119-121,177-180,662,*663-664*,683-693
			3: 129-130,212-228,238-238,262-393,409-413,418-420,428-430,459-475,511-593,601-638	1-3: 127-131,201-219,228-237,*238-239*,261,*262*,430-431,458-459,475-476,502-511,638-639
			4: 706-731	1-4: *697-702*,703-705,*706*,731-735

a. The involvement coefficients are calculated based on the structure of 1VOM and 1Q5G. See Table B.1 caption for details.

Table B.3
Results of dynamical domain partition and bending residues of 1Q5G for the normal modes with involvement coefficient (I_k) larger than 0.10 obtained by DynDom(84; 85)[a]

Mode[b]	I_k [c]	No. [d]	Range of domains [e]	Bending residues [f]
7	0.20	3	1: 3-486,505-688	2-1: *484*,485-487,502-504,*505-507*,508-509,683,*684-689*
			2: 487-504,689-695,736-745	
			3: 696-735	2-3: 695,*696*,*735-736*
8	0.39	5	1: 3-211,242-258,447-453,482-484,507-509,648-688	
			2: 698-731	
			3: 212-234,241-241,259-260,264-442,518-520,586-630,633-635	1-3: *200-201*,202-212,*241*,242,257-260,438-437
			4: 485-506,689-697,732-745	1-4: *484-485*,*506*,507,*688-689*
				2-4: *697-698*,699,731,*732*
			5: 235-240,261-263,454-481,510-517,521-585,631-632,636-647	1-5: 240-242,453-454,481-482,509-510,647-648
				3-5: 234-235,240-241,259-261,263,*264*,517-523,585-588,630-633,635-636
9	0.16	4	1: 86-95,99-106,117-118,123,125,163-194,199-490,504-655,677-683	
			2: 3-29,47-49,107-115,119-122,124,126-162,195-198,656-670	1-2: 105-107,115-119,122-124,*125*,126,*162*,163,194-195,198-199,*655*,656
				3-2: 28-30,*46-47*,49-50,*670-671*
			3: 30-46,50-85,96-98,671-675,684-691,693-694,696	1-3: 80-96,98-99,*675-677*,678,*681-685*
			4: 492-503,692,695,697	1-4: 483-492,*502-503*,504-505;3-4: *691-698*
12	0.28	6	1: 3-93,96-218,244-256,439-454,474-479,507-516,638-688	4-1: *218*,219-221,*222*,223,243-244,254-257,*258*,*437-438*,439,*454-455*,473-474,516-517,637-638
				3-1: 93-96,479,*480*,506-507,*688-689*
			2: 359-376,411-418	4-2: 356-359,*376-377*,408-411,*418-419*
			3: 94-95,480-506,689-697,734-745	
			4: 219-243,257-358,377-394,408-410,419-438,455-473,517-592,597-620,627-637	
			5: 395-407,593-596,621-626	4-5: 394-395,*407-410*,588-590,*591-593*,618,*619-621*,*626-627*
			6: 698-733	3-6: *697-699*,*733-734*
16	0.18	2	1: 18-40,44,47-78	
			2: 3-17,41-43,45-46,79-745	1-2: *78-79*
17	0.19	3	1: 356-420	2-1: *352-353*,354-356,*420-425*
			2: 3-32,46-48,80-355,425-488,503-688,690-692	
			3: 33-45,49-79	2-3: 32-33,43-44,*45-46*,*48-49*,78-80
21	0.19	2	1: 221-229,235-237,265-396,407-435,589-621,627-628	
			2: 399-405,459-476,512-513,517-585,623-626	1-2: 396-399,405-407,*585-589*, 621-627

a. The involvement coefficients are calculated based on the structure of 1Q5G and 1FMW. See Table B.1 caption for details.

Table B.4

Results of dynamical domain partition and bending residues of 1VOM for the normal modes with involvement coefficient (I_k) larger than 0.10 obtained by DynDom(84; 85)[a]

Mode[b]	I_k [c]	No. [d]	Range of domains [e]	Bending residues [f]
7	0.68	3	1: 697-724	3-1: 695-697, *724*, 725-727
			2: 3-487,503-688	3-2: 484-485, *486-487*, 488, *502-503*, *687*, 688-689
			3: 488-502,689-696,725-745	
8	0.22	3	1: 724-743	
			2: 3-696,744-745	1-2: *743-745*
			3: 697-723	1-3: *723-724*; 2-3: *695-697*
11	0.12	2	1: 3-93,95-108,116-124,138-157,671-684	
			2: 94,109-115,125-137,158-670,685-745	1-2: 93-95,108-109,115-116,*124-125*,137-138,157-158,670-671
12	0.12	3	1: 3-486,511-687	3-1: *486-487*,*510-511*,685-688
			2: 706-726	3-2: 704,*705*,706,726,*727*
			3: 487-510,688-705,727-745	

a. The involvement coefficients are calculated based on the structure of 1VOM and 1FMW. See Table B.1 caption for details.

Table B.5

Results of dynamical domain partition and bending residues of the crystal structures obtained by DynDom(84; 85)[a]

Structural pairs	No. [d]	Range of domains [e]	Bending residues [f]
1FMW vs. 1VOM	2	1: 3-485,502-689	
		2: 486-501,690-745	1-2: 485,*486*,*501-505*,506-509,689-690
1VOM vs. 1Q5G	2	1: 487-505,689-745	
		2: 3-486,506-688	1-2: *486-487*,505-510,681-683,*684*,685-686,*687*,688-689
1Q5G vs. 1FMW	2	1: 3-203,245-256,443-452,484-507,646-745	
		2: 204-244,257-442,453-483,508-645	1-2: 201-210,244-245,256-257,441-444,452-453,483-484,499-509,*645*,646

a. See Table B.1 caption for details.

Table B.6

Partition of hinge residues based on relations to SCA results and conservation[a]

Structural motif	Hinges	Appear in SCA	highly conserved ($\geq$ 80%)	The rest[b]
	19-25,27-30,35,44-50,78-87,95-96,99-101,109-110	81,82,95,110	99,109	19-25,27-30,35,44-50,78-80,83-87,96,100-101
β-sheet: 115-127	116-118,124-125		116,117,118	124-125
	154-155,162		154,162	155
β-sheet: 171-178				
P-loop: 179-186	183-186		183,184,185,186	
	193	193		
Loop 1: 199-218	200-201,218			200-201,218
	222		222	
Switch I: 233-238	238		238	
	239		239	
β-sheet: 240-262	241,258,262		241,262	258
	264,352-353,376-377,407-410,418-425,437-438	352,409,418,419	264,423,438	353,376-377,407-408,410,420-422,424-425,437
Switch II: 454-459	454-455		454,455	
Relay helix: 466-518	477-480,483-487,502-503,505-507,509-514	484,486,505,510,513,514	478,479,509	477,480,483,485,487,502-503,506-507,511-512
	585-589		587,588	585-586,589
Strut: 590-593	591-593	591,593		592
Loop 2: 610-628	619-621,626-627	627		619-621,626
	638		638	
β-sheet: 648-657	652-655	652	654,655	653
	660,663-664			660,663-664
SH1 helix: 669-690	670-671,675-677,680-690	688	675,676,680,683,686	670-671,677,681-682,684-685,687,689-690
	691-702,705,706	692	691,695,701	693-694,696-700,702,705,706
Converter: 710-747	723-724,727,732-736,743-745		743,745	723-724,727,732-736

a. The hinges include all the hinges in Table 2 (excluding those based on X-ray structures. The criterion for the highly conservation is set to 0.80. b. For the conservation of these residues, refer to Fig.B.1.

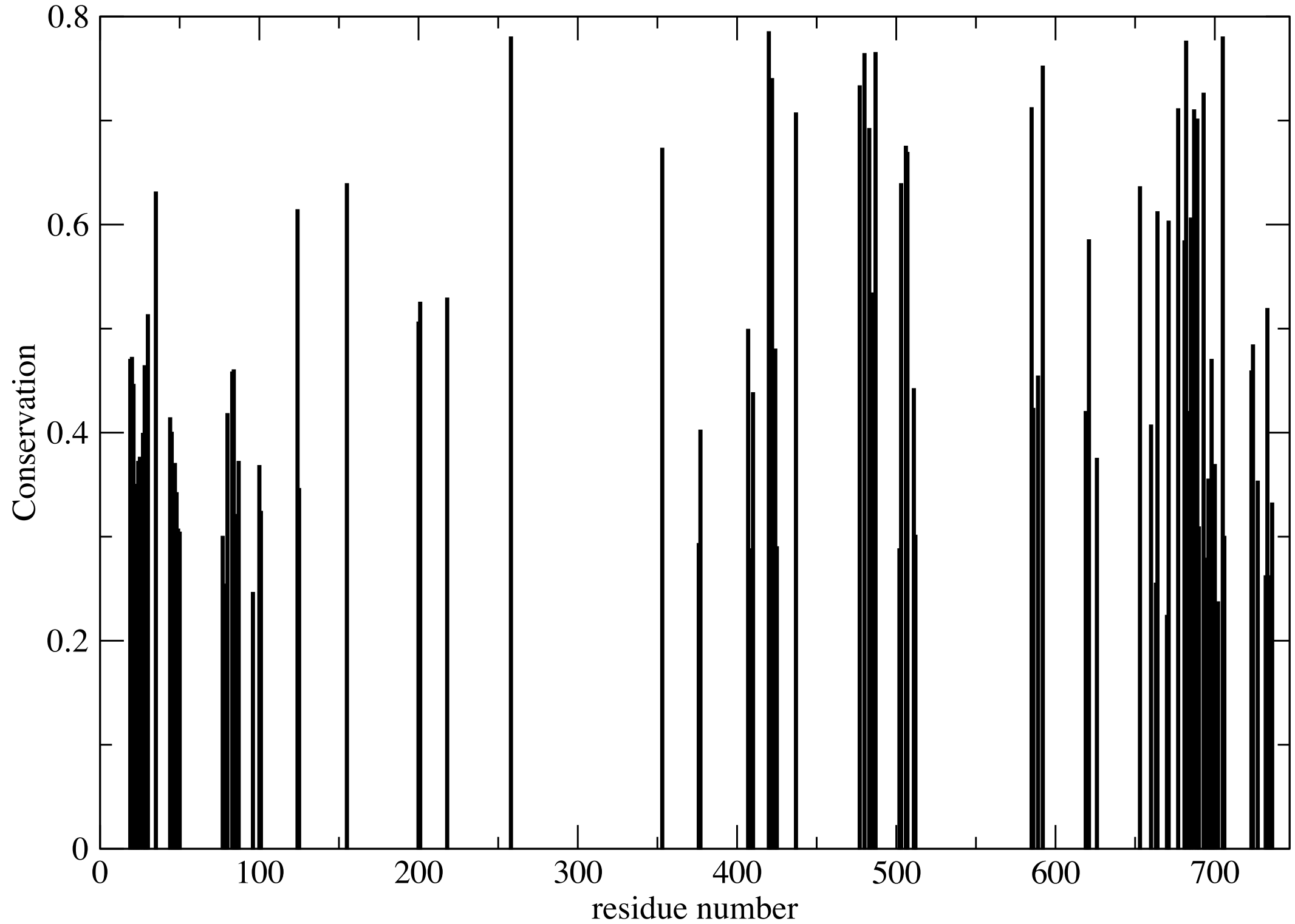

Fig. B.1: The degree of conservation for hinge residues that are neither overlapped with SCA core residues nor highly conserved (<80%); i.e., the last column in Table B.6.

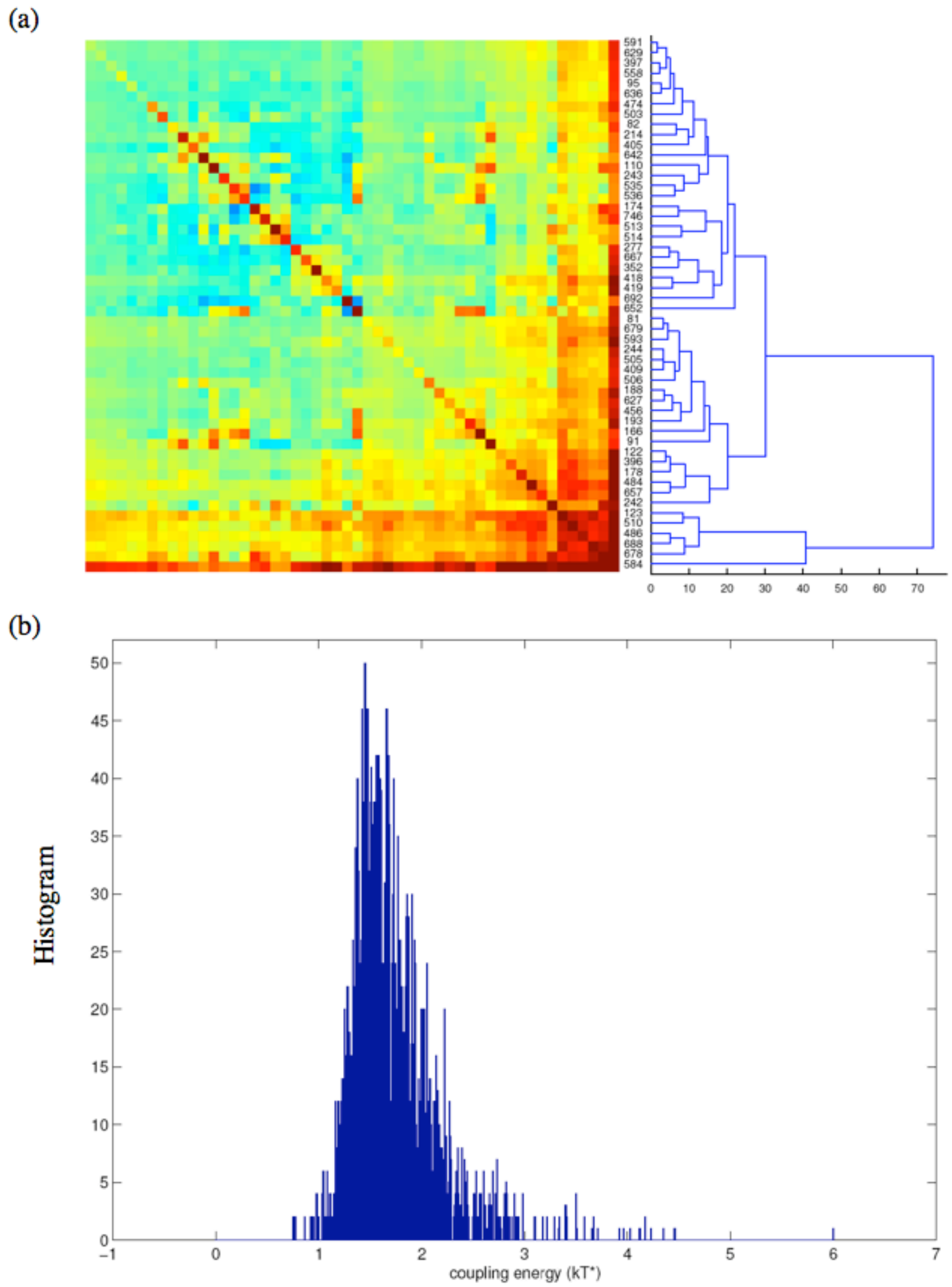

Fig.B.2: Results from the statistical coupling analysis associated with the 52 strongly coupled core residues. (a) The submatrix of pairwise coupling energies after hierarchical clustering, plotted with blue representing the lowest coupling energy (0 kT^*) and red representing the highest coupling energy (3 kT^*), respectively. kT^* is an arbitrary energy unit. Both columns and rows represent the residue number of *Dictyostelium* myosin motor domain. (b) The histogram of pairwise coupling energies, which shows a mean value of $1.72kT^*$, implying that these core residues are coupled to each other significantly.
